# The effect of anime character’s facial expressions and eye blinking on donation behavior

**DOI:** 10.1038/s41598-021-87827-2

**Published:** 2021-04-28

**Authors:** Hisashi Takagi, Kazunori Terada

**Affiliations:** 1 Graduate School of Engineering, Gifu University, 1-1 Yanagido, Gifu, 501-1193 Japan; 2 Faculty of Engineering, Gifu University, 1-1 Yanagido, Gifu, 501-1193 Japan

**Keywords:** Psychology, Human behaviour, Electrical and electronic engineering

## Abstract

Studies of the use of artificial agents and robots to solicit donations from people have suggested that the design of the agents must consider facial expressions. However, there has not been sufficient evidence to generalize the finding that the emotions conveyed by agents’ facial expressions can induce donations. In the present study, we conducted an experiment with an animated character that has intermediate realism and a different appearance from those in previous studies to replicate the finding that facial expressions represented by changes in the shapes of the eyes and mouth cause people to become more prosocial and to test whether we can extend this finding to the emotional expressions presented by changes in the dynamic properties of eyes. In the experiment, participants ($n=100$) played a hypothetical dictator game with an avatar that expressed its emotions by changing the shapes of its eyes, eyebrows and mouth and by changing the frequency of eye blinking. The results showed that the emotions expressed by changes in the shape of the facial parts contributed to eliciting a higher donation amount, consistent with previous studies. However, we could not find an additive effect of the emotional expression shown by eye blinking. The results suggest that, regardless of appearance, emotional expression is useful in the design of a virtual agent’s face, but it might not be necessary to consider the dynamic properties of the eyes.

## Introduction

Making a donation is a voluntary, nonrewarding activity, such as volunteering one’s time or donating financially^1^. With the development of the Internet society, the demand for donations via the Internet is increasing^2, 3^. In a future hybrid society of humans and machines, autonomous agents are expected to support interhuman prosocial behavior^4^. Charitable giving using ICT (information and communications technology) is a promising factor in promoting prosocial behavior. In recent years, research on agents and robots regarding soliciting donations has been conducted in the fields of HRI (human-robot interaction) and HAI (human-agent interaction). Some studies have suggested that faces could drive donations. Kim et al.^5^ applied the Hawthorne effect, in which being seen by someone motivates moral behavior, to the design of a robot’s appearance, showing that anthropomorphic robots with human-like faces promoted greater motivation to donate than robots with a functional appearance. A preliminary experiment in public spaces to induce donations suggested that a robot with glowing eyes and a spherical head was useful in inducing donations^6^. Another study suggested that the interactivity of the robot increased the amount of donations. Sarabia et al.^7^ conducted an experiment in which a robot with eye and mouth expressions on a tablet asked pedestrians to donate to Imperial Cancer Research and showed that the interactivity of the robot increased the amount of donations. Wills et al.^8^ conducted an experiment using a robot with a human-like head to raise money at a charity event to support people with ASD (autism spectrum disorder) and their families on a university campus, and they showed that the robot’s contingent behavior increased the amount of money raised. These studies suggest that we should consider not only the existence of faces but also behaviors, such as changes in facial expression, to design agents that motivate people to donate. Donations via the Internet are expected to increase in the future, and donations to virtual agents could become widespread in such cases. However, as far as we know, there has been no research on donations using virtual agents. In addition, there is no known relationship between emotional expressions through changes in an agent’s facial expressions and donations.

Psychological studies have shown that emotional expressions are a factor in modulating observers’ social behavior^9–12^. For example, studies have shown that expressions of joy increase the acceptance of offers in the ultimatum game^13^ and promote cooperation in the prisoner’s dilemma game^14^, whereas joy in negotiation can lead to inferences of leniency and low limits, which can encourage exploitation^15^. Expressions of regret after hostile decisions can lead to the restoration of cooperative relationships^16, 17^. The expression of anger leads others to make higher offers in the ultimatum game^18^ and increased concessions in negotiations since it signals to the observer that the expresser is tough and has ambitious limits^15, 16, 19^. On the other hand, inappropriate expressions of anger can cause negative reactions in the observer, leading to negotiation failure^20^. Emotional expressions can affect observer behavior because they indicate that the actor’s goal or concerns are satisfied or obstructed^21, 22^, leading observers to infer these internal states through a process of reverse appraisal^16, 23^. Some studies have used artificial agents to confirm the effects of emotional expression on prosocial behavior. Terada and Takeuchi^24^ used a software agent consisting of simple line drawings to show that the prosocial behavior of people changes as the angle of the mouth rises and falls in an ultimatum game. de Melo^16, 25–27^ used a virtual avatar that looks like a real person’s face and confirmed that people became more cooperative when the avatar showed cooperative emotional expressions compared to competitive emotional expressions in the prisoner’s dilemma. Kayukawa et al.^28^ replicated the findings of de Melo et al.^25^ with the emotional expressions of body movement and changes in eye color of a real humanoid robot, NAO. However, we still lack the evidence needed to generalize the findings that the emotional expressions of artificial agents are able to induce prosocial behavior, especially donations. One key feature that might affect prosociality is the realism or simplicity of the appearance of the agents. Therefore, in the present study, we focused on the faces of animated characters, which are intermediate between simple and real. The animated character used in the present study consisted of regions separated by edges and painted in a limited number of colors, and the texture of the clothes, hair, and skin as well as the reflection of light was expressed. In de Melo’s^16, 25–27^ study, the agent’s face was shown in a realistic 3D representation with shading. Compared with the agent in de Melo’s study, our agent has a flat face, flat mouth, and flat eyes and does not display realistic shadows and depth representation. Therefore, our agent might be simpler than the agent in de Melo’s study. In contrast, the agent in Terada’s^24^ study had eyes represented by simple true circles without pupils and a mouth represented by an arc. Therefore, our agents, which are portrayed in more detail, might be more realistic than the agent in Terada and Takeuchi’s study. Overall, the agent represented by line drawings used by Terada and Takeuchi might be at the extreme end of simplicity, and the human-like avatars used by de Melo are at the extreme end of realism; thus, we defined the agent used in the present study as that with an intermediate appearance between simple and real.

One aspect of the facial expressions of agents that could make people more prosocial is the dynamic properties of the eyes. Social psychological research has shown that the presence of static eyes makes people more prosocial. Haley and Fessler^29^ showed that the presence of eye images in the dictator game increased the mean number of donations. Since the work of Haley and Fessler^29^, there have been many studies on the impact of eye presence on prosocial behavior. In the study by Bateson et al.^30^ examining the effects of eye imagery, participants paid almost three times as much for a beverage charge collection box set up in a university coffee lounge if there was an eye image. Powell et al.^31^ conducted a survey of donation boxes with eye images in public places and showed that there was a significant increase in the amount donated in the presence of eyes compared to none. However, Nettle et al.^32^ reported conflicting results for the mean increase in donation amounts. Nettle’s meta-analysis revealed that, although there have been studies showing an increase in the amount of donations^29, 33, 34^, there have also been studies that did not replicate these findings^35–37^, indicating the need for further studies. One feature that can be implemented in agents to promote prosociality but that has not been considered in psychological experiments is eye movements. While the above study investigated the impact of static “eye presence” on donation behavior, only a small number of studies have focused on the dynamic properties of the eye^38–40^. Prochazkova et al.^38^ conducted an fMRI study using trust games and found that pupil changes in interacting partners were mimicked, consequently promoting trust. Ozeki et al.^39^ showed that tracking a person’s decision-making process with the agent’s eyes does not increase donation behavior. However, empirical studies with agents have predicted that other dynamic properties of the eyes, such as blinking, increase prosociality. Weibel et al.^40^ manipulated an avatar’s appearance to investigate how participants perceived the avatar. They investigated the pupil size, eye blinking frequency, and viewing angle of avatars. The results showed that avatars with less frequent eye blinking and larger eyes were rated as more attractive and sociable. Furthermore, Maffei and Angrilli^41^ showed that high-frequency blinks encoded negative emotions, and low-frequency blinks encoded positive emotions. As described above, studies have confirmed that emotional expressions elicit a person’s sociability^24, 25^. Considering these findings together, we hypothesize that, if the frequency of eye blinking encodes emotional states, then it elicits a person’s prosociality. Therefore, in the present study, we focused on the frequency of eye blinking as one of the dynamic properties of eyes.

In the present study, we investigated whether the eye blinking and facial expressions of an animated character affect people’s donation behaviors. We used an experimental paradigm that was proposed by Terada and Takeuchi^24^ and was used in a replication study conducted by Ozeki et al.^39^, in which the agent’s facial expression changes according to the position of a slider bar that indicates the amount of the donation. Participants were given one minute to decide on the amount of the donation and were allowed to move the slider bar during that time.

## Methods

### Participants

We determined the sample size before the start of data collection based on a power analysis. The $G^*Power 3.1.6$^42^ analysis (effect size f $= 0.29$, $\alpha = 0.05$ (two-tailed) and $1-\beta = 0.80$) suggested an initial target sample size of $N = 96$. One hundred participants (80 male, 20 female, $M_{age}=43.96$, $SD_{age}=10.25$) participated in the experiment. Two participants were excluded due to failure to complete the survey, and a final sample size of 98 participants was obtained. All of the participants were recruited through Yahoo! Crowdsourcing (crowds) and received Yahoo! points as a reward only if they answered all of the questions. Recruitment in the cloud was conducted on September 5, 2019. Participants participated in the study by giving online informed consent. The experiments were undertaken in compliance with national legislation and the Code of Ethical Principles for Medical Research Involving Human Subjects of the World Medical Association (Declaration of Helsinki).

**Figure 1:**
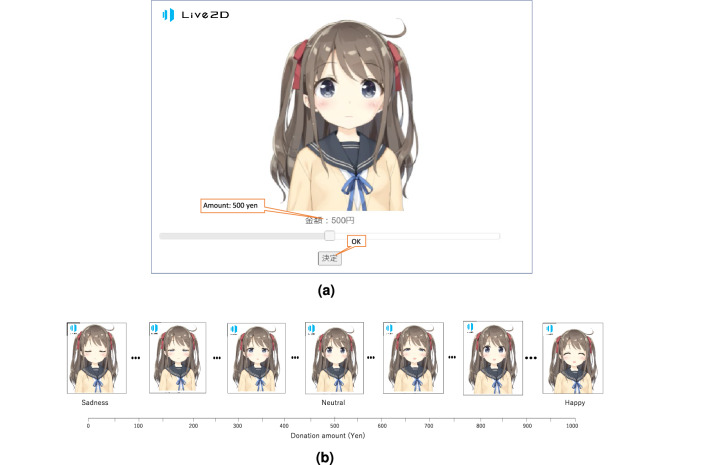
User interface and changes in the facial expression of the avatar. The anime character imagery figures were obtained from Live2D Cubism, Live2D Inc.; all rights reserved. (**a**) User interface. The facial expressions of the avatar and the amount of the donation change according to the position of the slider bar. If the slider bar goes to the left end, the amount is 0, and the avatar expresses sadness. If the slider bar goes to the right end, the amount is the maximum value, and the avatar is happy. In the middle, the amount is the mean, and the avatar is neutral. (**b**) Changes in the facial expression.

### Materials

We created a web interface with an avatar and a slider bar (Fig. 1a). The users of the interface are able to move the slider bar. The facial expressions of the avatar and amount of the donation change according to the position of the slider bar. If the slider bar is moved to the left end, the amount is 0, and the avatar expresses sadness. If the slider bar is moved to the right end, the amount is the maximum value, and the avatar is happy. In the middle, the amount is the mean, and the avatar is neutral. The facial expressions change smoothly in response to the movement of the slider bar. We used a “Hiyori Momose”, which is a Japanese anime-style female figure with large eyes and is included in the software Live2D Cubism from Live2D Inc. Although the amount of the donation changed in increments of fifty yen, the smallest unit, depending on the position of the slider bar, the facial expressions were presented in 21 discrete levels. We divided the positions of the slider bar into 21 steps at equal intervals. Each of the 21 facial expressions shown in Fig. 1b were created as a still image file (without blinking) or a movie file (with blinking) by manipulating the parameters of the eyes, eyebrows, and mouth in “Live2D Cubism” (for details, see Supplementary Fig. S1 and Table S1). The two levels of facial expression were static and changing. At the static level, a neutral expression was shown regardless of the position of the slider bar. At the changing level, the facial expression was changed according to the position of the slider bar as described above.

It is known that eye blinking usually occurs, on average, once every 3 s^43^. To prevent the surface of the eye from drying out, blinking every 30 s to a minute is sufficient. Thus, physiological factors alone cannot explain all eye blinking. Eye blinking can be classified as voluntary and or involuntary^44^. The former can be controlled by the person, while the latter is an uncontrollable behavior. Furthermore, the latter can be classified as reflexive or spontaneous. Spontaneous blinking is a reflex-like behavior that occurs in the absence of an eliciting stimulus (external factors, such as an incoming projectile). Maffei and Angrilli^41^ showed that high-frequency blinks encode negative emotions, and low-frequency blinks encode positive emotions. They presented a perceptual task (with video clips) categorized into six emotional categories and showed that changes in the blinking rate were associated with emotions. The rate of blinking decreased in categories such as joy (three blinks per min) and increased in categories such as fear and sadness (24 blinks per min). In the present study, the frequency of eye blinking was designed with reference to Maffei’s^41^ study. The two levels of eye blinking were (1) without blinking and (2) with blinking, and we investigated the effects of eye blinking. In the levels with blinking, the blinking rate was set to an average of 16 blinks per minute in the initial state (50:50 allocation). The blinking rate was set to an average of three blinks per min when happy (maximum amount) and to an average of 24 blinks per min when sad (minimum amount). At the unblinking level, the avatar had either open or closed eyes. In the case without blinking, the images were output as still images (PNG format), and in the case with blinking, they were output as animation (APNG format). We created two images with different blink timings without changing the blink frequency, and we randomly displayed one of them.

### Procedure and measures

First, the participants were asked to imagine a situation in which they received a windfall of 1000 yen, and then they were asked to donate to children orphaned by traffic accidents. The participants moved the sliding bar below the avatar to determine the donation amount. To determine the final donation amount, the participants had to press a button that corresponds to OK (Fig. 1a). Participants received reward points regardless of the total amount of their donations. Next, they were asked to complete the items on the Godspeed questionnaire^45^. We used the Godspeed questionnaire, which is a standardized measure to assess anthropomorphism, animacy, likeability, perceived intelligence, and perceived safety in human robot interaction. Although the questionnaire is intended to assess interactions with robots, we used it to explore mediated factors that affect donation other than the dynamics of eyes. The answers to the questionnaire were given on a five-point Likert scale. This procedure was approved by the Ethics Committee of Gifu University. All of the experimental methods used were approved by the Medical Review Board of the Gifu University Graduate School of Medicine (IRB ID# 2019-092).

### Data analysis

The experiment followed a $2\times 2$ (facial expression [static, changing] $\times$ eye blinking [with blinking, without blinking]) between-participants factorial design. All of the statistical analysis were performed using SPSS (IBM SPSS Statistics 26). For all of the statistical tests, a significance threshold of $p<0.05$ was adopted. The partial eta squared ($\eta {_p}^2$) was used as a measure of the effect size^46^. $R^2$ was used as a measure of the effect size in multiple regression analysis^46^.

## Results

**Figure 2:**
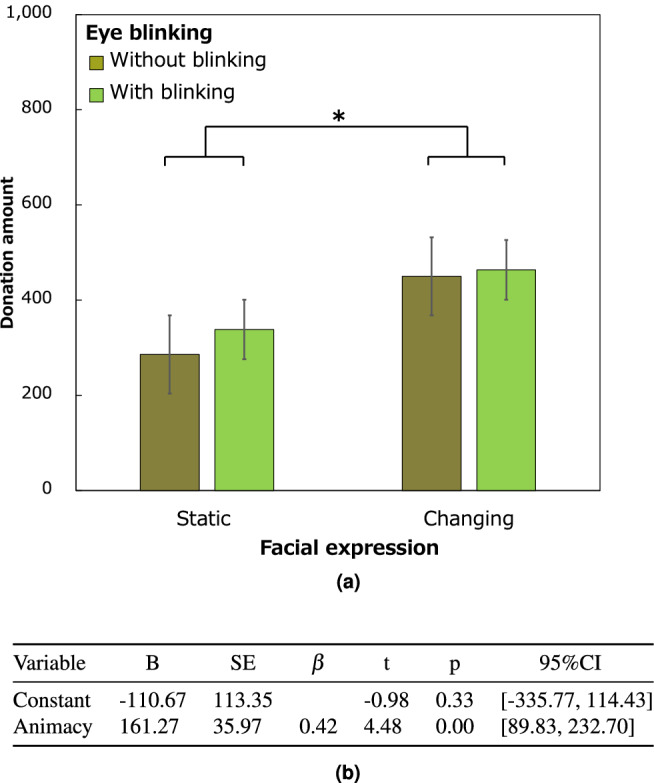
Results. (**a**) Participant donation rate. The error bars indicate standard errors. *$p < 0.05$. (**b**) Multiple regression.

Figure 2a shows the mean and standard error of the participants’ donations. There was no significant interaction between blinking and facial expression $(F(1, 94) = 0.10, p = 0.75, \eta {_p}^2 = 0.001)$. The results revealed that the ratings of the donation were significantly higher $( F(1, 94) = 5.69, p = 0.019, \eta {_p}^2 = 0.06)$ in the changing facial expression condition $(M = 456.4, SD = 320.0)$ than in the static condition $(M = 312.8, SD = 274.4)$, and there was no significant main effect of blinking $(F(1, 94) = 0.3, p = 0.59, \eta {_p}^2 = 0.003)$. The mean durations spent deciding the amount of the offer did not differ across conditions (for details, see Supplementary Table S2).

A two-way ANOVA was performed for each of the five concepts in the Godspeed questionnaire (anthropomorphism, animacy, likability, safety, and intelligence), and none of them showed a significant difference between the interaction and the main effect. Then, we conducted a multiple regression analysis using the stepwise method. A multiple linear regression was performed to predict donations based on the five concepts.

A significant regression equation was found $( F(1, 96) = 20.09, p = 0.00 )$ with an $R^2$ of 0.17. Only animacy was a significant predictor of donations. The participants’ predicted donations were equal to $-110.67 + 161.27$ (animacy), where animacy is coded on a five-point Likert scale. Participant donations increased by 161.27 yen for each increment in animacy (Fig. 2b).

## Discussion

In the present study, we investigated whether the eye blinking and facial expressions of an avatar affect donation behavior. The results showed that participants donated more to children orphaned by traffic accidents when the avatar’s facial expression changed in accordance with their exploratory action before the final decision on the amount of the donation, whereas a change in eye blinking did not affect the amount of the donation. The results indicated that changes in facial expressions make people to become more cooperative, in line with previous studies^16, 24–27^. Terada and Takeuchi^24^ showed that the emotional expressions in a line drawing of an artificial agent contributed to increasing the amount of money offered in an ultimatum game. de Melo^16, 25–27^ showed that changes in the facial expressions of a virtual agent with a human-like appearance induced cooperative behavior in humans in the prisoner’s dilemma. In the present study, a Japanese anime-style female figure with large eyes was used as an avatar. Taken together, these studies suggest that, regardless of the differences in the game and the appearance of the avatar, people may become more cooperative when avatars display emotional expressions.

In the present study, we predicted that not only changes in facial expression but also changes in eye blinking promote donation behavior. This prediction was based on an assumption that changes in avatars’ blinking rates also code the emotional states of the avatar. This assumption was based on Maffei’s^41^ findings, in which a participant’s blinking rate increased when he/she was watching video clips that were categorized as sad or frightening and decreased when the video clips were categorized as joyful. We set the avatar’s blink rate to range from 3 times per minute on average when the maximum amount was offered to 24 times per minute on average when the minimum amount was offered, and the blinking rate varied according to the position of the slider bar that determined the amount of the donation. However, the results did not support our predictions. There are at least two possible reasons why a change in the blink rate did not affect donation behavior. First, it is possible that the range of the blink rate was not appropriate for representing emotional states. In present study, we selected the blink rate according to the study of Maffei and Angrilli^41^. According to the study conducted by Weibel et al.^40^, avatars that blinked less often were rated as more attractive within a blinking rate range of 16–60 times per min. The range of 3–24 times per min used in the present study is narrower and lower than that of Weibel. It is possible that a wider range of blinking rates could code the avatar’s emotional states and induce cooperative behavior in the observer of the avatar. Second, the social effects of vitality-mediated blinking may not have been fully realized because the avatar was female. In the present study, the regression analysis of the Godspeed questionnaire showed that animacy mediated the participants’ donation behavior. In the Godspeed questionnaire^45^, animacy includes semantic differentials, such as dead/alive and stagnant/lively. Thus, we can say that animacy is closely related to active energy, including vitality. Takashima et al.^47^ showed that the vitality perceived from eye blinking is stronger for male characters than female characters. These results indicate that our female avatar was not able to express vitality by eye blinking; therefore, it did not affect donation behavior. Further studies to confirm the connections among eye blinking, animacy, and emotional states should be performed. In the present study, we used an agent with large eyes, which is a typical characteristic of Japanese animated characters. It is possible that blinking with normal sized eyes, not exaggerated eyes, is able to extract prosocial behavior from people. This possibility should be tested.

On the other hand, the present study replicated the study conducted by Ozeki et al.^39^, in which the eye movements of a virtual agent had no effect on donation behavior. They showed that the eye movements of a virtual agent following a mouse cursor, which indicated that the agent was watching the decision of the participants, did not affect the donation amount^39^. Together with Ozeki et al.’s study, it is possible to draw the general conclusion that the dynamic properties of the eyes of virtual agents do not affect cooperative behavior. However, as discussed in the previous paragraph, a detailed investigation of the effects of eye dynamics on cooperative behavior should be conducted. Social psychological research has shown that the presence of static eyes makes people more prosocial^29–31^. In the present study, we used the static eyes without blinking as a baseline; therefore, we did not directly investigate the effect of the presence of eyes. Thus, our results are not generalizable to the presence of dynamic eyes.

Although the influence of embodiment was not investigated in the present study, some predictions could be obtained from the previous literature. Lee et al.^48^ showed that people evaluate a physically embodied social agent more positively than a disembodied social agent and that the feeling of social presence is a key mediating variable for the effects of physical embodiment on the evaluation of a social agent. Bainbridge et al.^49^ showed that people are more compliant to unusual requests from a physically present robot than a video-displayed robot, suggesting that greater trust is afforded in the case of physical presence. From these studies, we would expect that embodied robots would have a more positive social presence and trust than virtual agents; therefore, embodied robots would induce a larger donation than virtual agents. Correia et al.^50^ showed that, when the agents were disembodied, the prosocial agent was rated more positively, and the selfish agent was rated more negatively compared to when they were embodied, suggesting that selfish behavior could be masked by the presence of the body. This implication suggests that embodied agents are more likely to extract profits from people than virtual agents, in turn suggesting that they might be able to extract more donations from people. Connolly et al.^51^ showed that emotional reactions presented by a group of bystander robots could motivate people to perform prosocial behaviors. The virtual agent in the present study was not the recipient of the donation, and in this sense, it was a bystander. The results of Connolly et al.^51^ and our study could be generalized to a finding that the behavior of bystander agents could promote people’s prosocial behavior. However, more detailed research must be conducted.

There are some limitations to present study. First, the participants were a culturally homogeneous group, and the gender ratio was not specifically controlled for in this analysis. Culture and gender can influence the expression and interpretation of emotional expressions^39, 52^. It is necessary to use a more diverse sample to study the gender and cultural influences in human-agent cooperative behavior mediated by emotional expressions. Second, a study investigating android-human interactions conducted by Takatsu et al.^53^ showed that eye contact is associated with blink synchronization. We did not address blink synchronization and gazing between the agent and participants. The interactive properties of eye movement require further investigation. Third, various appearances should be investigated. In the present study, the virtual agent was a Japanese anime-style female avatar with large eyes. In the study by Terada et al.^54^, a comparison of characters, such as people, dogs, and Buddha images, was performed. The results of the comparison showed that dog-shaped and humanoid avatars are more effective in inducing purchases than Buddhist statues and realistic persons. Comparisons with agents of various types and appearances, such as variations in gender; the presence or absence of animated expressions, such as eye size; and nonhuman characters, such as animals and robots, are needed for more generalized findings. In the present study, we employed a hypothetical dictator game, in which no real money is exchanged. Ben-Ner et al.^55^ showed that there is no difference in the amount of money offered in the hypothetical dictator game and real dictator game. From this study, it is suggested that people will be likely to donate the same amount of money as in the present study, even when they spend real money. However, a study by Xu et al.^56^ indicated that the information processing in the brain regarding risk taking might be different between real and hypothetical monetary rewards. Therefore, further study with real money is needed.

## Supplementary information

Supplementary Information 1.

Supplementary Information 2.

Supplementary Video 1.

Supplementary Video 2.
