# Supplementary material for: The effect of anime character’s facial expressions and eye blinking on donation behavior
Source: Sci Rep. 2021 Apr 28;11:9146. doi: 10.1038/s41598-021-87827-2 (PMC8080783; doi:10.1038/s41598-021-87827-2)
Supplement: Supplementary file 1 — Supplementary Information 1. [file 41598_2021_87827_MOESM1_ESM.docx]

**Supplementary Information**

**The effect of anime character's facial expressions and**

**eye blinking on donation behavior**

**Hisashi Takagi** ^a^ **and Kazunori Terada** ^b^

^a^ Graduate School of Engineering, Gifu University, 1-1 Yanagido, Gifu 501-1193, Japan

^b^ Faculty of Engineering, Gifu University, 1-1 Yanagido, Gifu 501-1193, Japan

**
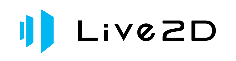
**
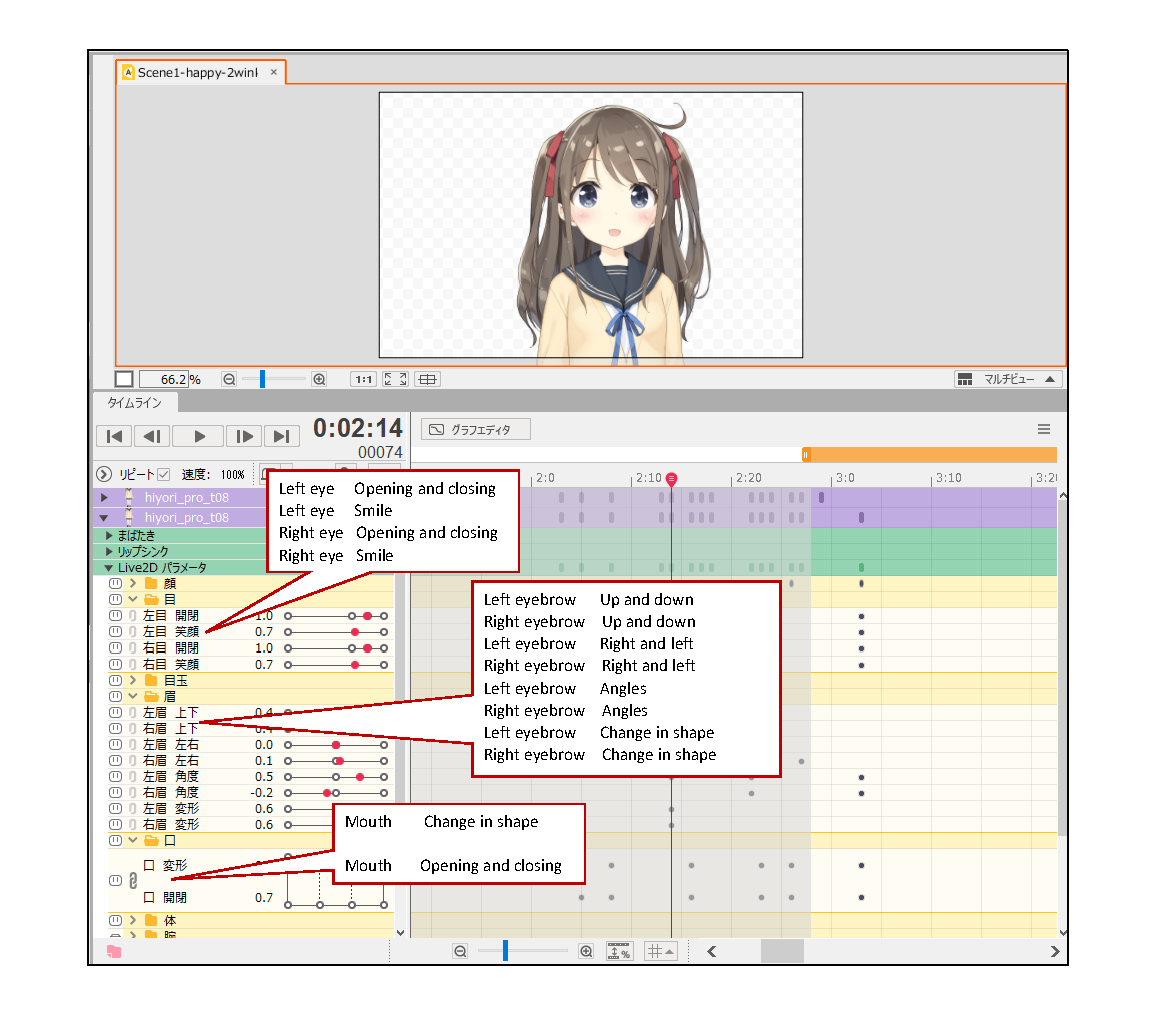


**Supplementary Figure S1.** Interface of Live2D Cubism, which is an editor for the facial expressions of animated characters. The imagery figure of the anime character and the software interface were obtained from Live2D Cubism, Live2D Inc.; all rights reserved.

**Supplementary Table S1.** Parameters of Live2D software for emotional expressions. The number in parentheses is the number of the face in Figure 1b. The parameters of the saddest, neutral, and happiest face are shown in the table. The other intermediate facial parameters were fine-tuned by the experimenter after careful consideration to ensure that the facial expression changed equally as the step changed.

| Parameters |  |  | Sad expression | Initial situation | Happy expression |
| --- | --- | --- | --- | --- | --- |
| Eyes | Left eye | Opening and closing | 0 | 1 | 1.1 |
|  | Left eye | Smile | 0 | 0 | 1 |
|  | Left eye | Opening and closing | 0 | 1 | 1.1 |
|  | Right eye | Smile | 0 | 0 | 1 |
| Eyebrows | Left eyebrow | Up and down | -0.8 | 0 | 0.3 |
|  | Right eyebrow | Up and down | -0.8 | 0 | 0.3 |
|  | Left eyebrow | Left and right | -0.2 | 0 | 0 |
|  | Right eyebrow | Left and right | -0.2 | 0 | 0 |
|  | Left eyebrow | Angles | 0.4 | 0 | 0.3 |
|  | Right eyebrow | Angles | 0.4 | 0 | 0.1 |
|  | Left eyebrow | Change shape | -0.4 | 0 | 0.6 |
|  | Right eyebrow | Change shape | -0.4 | 0 | 0.6 |
| Mouth | Mouth | Change shape | -1.5 | 1 | 0.9 |
|  | Mouth | Opening and closing | 0.1 | 0 | 0.8 |

**Supplementary Table S2.** Mean and standard deviation of durations spent deciding the amount of the offer (seconds). A two-way ANOVA revealed that there was no significant interaction between facial expression and blinking $(F\left( 1,94 \right)=0.15,p=.699, \eta_{p}^{2}=.002)$, and no significant main effect of facial expression $(F\left( 1,94 \right)=0.16,p=.694, \eta_{p}^{2}=.002)$; or blinking $(F\left( 1,94 \right)=0.01,p=.976, \eta_{p}^{2}=.000)$.

|  | | | Facial expression | |
| --- | --- | --- | --- | --- |
|  |  |  | Changing | Static |
| Blinking | With blinking | Mean | 163.2 | 163.4 |
|  |  | SD | 69.9 | 77.0 |
|  | Without blinking | Mean | 156.1 | 169.5 |
|  |  | SD | 73.9 | 110.3 |

**Supplemental Movie S1.** Avatar's blink. The condition of blinking is high, neutral, or low. The conditions are blinking with no change in expression. The anime character imagery figures were obtained from Live2D Cubism, Live2D Inc.; all rights reserved. Facial_Expression_Without_Blinking.mp4

**Supplementary Movie S2.** Facial expression changes in the avatar. Each stage of facial expression change. The anime character imagery figures were obtained from Live2D Cubism, Live2D Inc.; all rights reserved. Static_With_Blinking.mp4
